# A Prospective Study on the Outcome After Mass Closure of Post-laparotomy Wound Dehiscence in a Tertiary Care Hospital, Tamil Nadu, India

**DOI:** 10.7759/cureus.59642

**Published:** 2024-05-04

**Authors:** Japhereena Murugavel, Arasu Vajiravelu Thirunavukkarasu, Vinoth Gnana Chellaiyan, Vijayalakshmi Sridharan

**Affiliations:** 1 Department of General Surgery, Government Peripheral Hospital, Stanley Medical College, Chennai, IND; 2 Department of General Surgery, Chengalpattu Medical College, Chengalpattu, IND; 3 Department of Community Medicine, Chettinad Hospital and Research Institute, Chettinad Academy of Research and Education, Chennai, IND; 4 Department of Community Medicine, Chettinad Hospital and Research Institute, Chennai, IND

**Keywords:** post-laparotomy, wound healing, mass closure, reclosure, wound dehiscence

## Abstract

Introduction

The ideal abdominal wound closure provides strength and a barrier to infection. The major cause of morbidity following any laparotomy is abdominal wound dehiscence. For prompt patient recovery and outcome factors influencing wound healing following mass closure of post-laparotomy, wound dehiscence patients are evaluated in this present study. The aim of the study was to evaluate the outcome and various complications following mass closure of post-laparotomy wound dehiscence.

Materials and methods

A prospective study was conducted among 50 patients admitted to the Department of General Surgery, Tamil Nadu, India, with wound dehiscence following emergency and elective laparotomy surgeries managed with mass closure during the study period from 2021 to 2022. The chi-square test and Fischer’s exact test were done.

Results

Mass closure of post-laparotomy wound dehiscence was more common among males (74%, n=37) and less common in the age group 20-30 years (12%, n=6). Prolonged bleeding time and clotting time post-surgery were associated with the type of surgery with a significant p-value of 0.007 and 0.001, respectively, by Fischer’s exact test. The presence of urine albumin was also associated with the type of surgery with a significant p-value of 0.02. Surgical site infection (postoperative complication) was associated with the type of surgery and operating time with a significant p-value of 0.004 and 0.03, respectively.

Conclusion

Abdominal wound dehiscence is a serious and challenging postoperative complication that necessitates immediate intervention. Strict postoperative care places emphasis on reducing the risk of wound infection and other factors related to wound dehiscence.

## Introduction

Wound dehiscence after abdominopelvic surgery is a rare but significant complication of general, transplant, obstetric, gynecologic, and plastic procedures. Abdominal surgical wound dehiscence occurs up to 3% of the time, according to research associated with 25% mortality [1,2]. One of the most crucial elements that contribute to the success of an operation is the quick and painless healing of surgical wounds. Patients who are undergoing laparotomy dehiscence are linked with high rates of morbidity and mortality. Abdomino-pelvic surgery complications like this can result in prolonged hospital stays, higher healthcare costs, and higher mortality rates [3]. Additionally, there is evidence linking abdominal wound dehiscence to poor quality of life, incisional hernia incidence, and bad body image [4,5].

Abdominal wound dehiscence remains a prominent cause of morbidity following any laparotomy, whether elective or emergency. Thirty abdominal fascial wound dehiscence occurred in 2,761 patients undergoing major abdominal surgery during five years (1%) through multivariate analysis [6]. Abdominal wound dehiscence produces severe adverse effects. Research activities focussed on figuring out risk factors for this complication. Age, gender, diabetes, hypertension, obesity, use of steroids, infection, hemodynamic instability, and malignancy are all associated with abdominal wound dehiscence, according to several studies. Research has examined the efficacy of risk assessments based on these characteristics to identify patients who are more likely to develop wound dehiscence and to direct clinical therapy to help prevent this complication following abdominopelvic surgery [7,8].

Layered closure is described as the separate closure of the individual component of the abdominal wall, specifically the peritoneum and distinct musculoaponeurotic layers, whereas mass closure is the closure of all layers of the abdominal wall (except the skin) as a single structure. Several studies conducted to prove that mass closure is a simple technique with a lower incidence of wound dehiscence following abdominal wound closure. Although identifying risk factors may help avoid postoperative abdominal wound dehiscence, there has not been much research done to analyze the variables that influence how laparotomy wounds heal after dehiscence. The study aimed to evaluate the outcome and complications following mass closure of post-laparotomy wound dehiscence.

## Materials and methods

Study design: The study was of a cross-sectional type.

Study site: A tertiary care hospital Department of General Surgery in the Chengalpattu district of Tamil Nadu, India, was the site of the study.

Study period: The study was conducted as a prospective study from April 2021 to March 2022.

Sample size: Data were calculated and analyzed using IBM SPSS Statistics (version 22.0). Normality and variable association were examined using chi-square or Fischer’s exact test. After calculating the sample size, it was determined that 50 people (including a 10% non-response rate) would need to be incorporated to accurately measure the 3% prevalence of wound dehiscence following laparotomy, with 5% absolute precision at a 95% confidence interval and 80% power [9].

Study population: All the patients who underwent mass closure following (elective and emergency) post-laparotomy wound dehiscence in the Department of General Surgery in a tertiary care hospital after abiding by inclusion and exclusion criteria during the study period.

Inclusion criteria: (1) patients admitted under the Department of General Surgery who underwent mass closure following laparotomy procedure; (2) patients aged 20-70 years; (3) patients who are willing to participate in the study with informed written consent; and (4) patients who consented to follow-up for four weeks after mass closure.

Exclusion criteria: (1) patients age group less than 20 years and more than 70 years; (2) patients with incomplete records or missing data on the date of wound onset; (3) use of mesh during any surgical procedure following dehiscence; and (4) patients who are not willing to participate or not willing to sign the consent.

Study conducted: The study was carried out prospectively. Included in the questionnaire were age, gender, comorbidities, and type of surgery. They have undergone thorough clinical examinations and routine pre-operative investigations. We recorded the duration of abdomen closure for each instance. These data are of utmost importance as they help us ensure that all procedures are performed proficiently and with due diligence. After surgery, all patients were given intravenous antibiotics for two to three days, followed by oral antibiotics for five to seven days. The agents used are antimicrobials; cefotetan, cefoxitin, imipenem, piperacillin-tazobactam, and ticarcillin-clavulanic acid have all been shown to be effective in treating as monotherapy, while ceftolozane-tazobactam was used in combination with metronidazole. The use of antibiotics was continued beyond 10 days whenever indicated. The wound status was evaluated on days three, five, seven, and 10 following the suturing procedure, and its condition was documented. The Centers for Disease Control and Prevention created a surgical wound classification system (SWC: I, clean; II, clean/contaminated; III, contaminated; and IV, dirty) to preemptively identify patients at risk of surgical site infection (SSI). Postoperative complications such as SSIs were recorded and appropriately managed. Exuding wounds were cultured and microscopically examined.

## Results

The mean age of the study participants is 43.06 ± 13.46, ranging from 20 to 70 years (Table 1). It is evident that mass closure of post-laparotomy wound dehiscence was more common among males (74%, n=37) and less common in the age group 20-30 years (12%, n=6). Mass closure of post-laparotomy wound dehiscence was more common among patients undertaken for emergency procedures. Around 88% (n=44) of the patients who presented with wound dehiscence underwent emergency procedures. Among the emergency procedures, obstruction was most common (36%, n=18). All the patients who developed wound dehiscence following elective procedures had a history of malignancy. Of the surgical procedures, wound dehiscence was high in ostomy (28%, n=14), followed by patch closure (24%, n=12) and resection anastomosis (22%, n=11).

**Table 1:** Baseline characteristics of the study population n=number of study participants

Variables	n	%
Age (years)		
20-30	6	12
31-40	11	22
41-50	13	26
51-60	8	16
61-70	12	24
Gender		
Female	13	26
Male	37	74
Indication for surgery		
Perforation	15	30
Obstruction	18	36
Blunt injury	11	22
Malignancy	6	12
Surgical procedure		
Patch closure	12	24
Resection and anastomosis	11	22
Ostomy	14	28
Gel foam packing	3	6
Splenectomy	2	4
Bladder repair	4	8
Appendicectomy	2	4
Gastrectomy-subtotal	1	2
Others - Whipple’s procedure	1	2

In around 88% (n=44) of the cases, suture removal was done on postoperative day (POD) 14 after mass closure of post-laparotomy wound dehiscence, while in 6% (n=3) of patients, suture removal was done on POD 20. Table 2 shows that comorbidities such as obesity, diabetes mellitus, hypertension, chronic obstructive pulmonary disease (COPD), dyslipidemia, and smoking were not associated with the type of surgery in our study population. From Table 3, it is evident that prolonged bleeding time and clotting time post-surgery are associated with the type of surgery, with a significant p value of 0.007 and 0.001, respectively by Fischer’s exact test. The presence of urine albumin was also associated with the type of surgery, with a significant p value of 0.02. Other lab parameters such as hemoglobin, blood urea, serum creatinine, and random blood glucose were not associated with the type of surgery and so were X-ray changes.

**Table 2:** Association between comorbidities and the type of surgery with wound dehiscence (n=50) n=number of study participants *Dyslipidemia - cholesterol > 200 mg/dL A chi-square test or Fischer’s exact test was done, and a p-value <0.05 was considered significant.

Comorbidities	Type of surgery		P value
	Emergency, n (%)	Elective, n (%)	
BMI > 30 (kg/m^2^)			
Yes	12 (26)	1 (25)	0.962
No	34 (74)	3 (75)	
Diabetes mellitus			
Present	10 (21.7)	1 (25)	0.88
Absent	36 (78.3)	3 (75)	
Hypertension			
Present	11 (23.9)	0	0.268
Absent	35 (76.1)	4 (100)	
COPD			
Present	10 (21.7)	1 (25)	0.88
Absent	36 (78.3)	3 (75)	
Dyslipidemia*			
Present	10 (21.7)	1 (25)	0.88
Absent	36 (78.3)	3 (75)	
Smoking			
Yes	13 (28.3)	0	0.216
No	33 (71.7)	4 (100)	

**Table 3:** Association between investigation and the type of surgery with wound dehiscence (n=50) n=number of study participants A chi-square test or Fischer’s exact test was done, and a p-value <0.05 was considered significant.

Postoperative investigation		Type of surgery		P value
		Emergency, n (%)	Elective, n (%)	
Hb	> 11 g/dL	32 (69.6)	3 (75)	0.82
	< 11 g/dL	14 (30.4)	1 (25)	
BT	2-9 min	30 (65.2)	4 (100)	0.007
	>9 min	16 (34.8)	0	
CT	Normal	35 (76.1)	1 (25)	0.001
	Abnormal	11 (23.9)	3 (75)	
Blood urea	<40 mg/dL	39 (84.8)	2 (50)	0.082
	>40 mg/dL	7 (15.2)	2 (50)	
Serum creatinine	>2 mg/dL	40 (87)	3 (75)	0.509
	<2 mg/dL	6 (13)	1 (25)	
RBS	>200 mg/dL	31 (67.4)	3 (75)	0.754
	<200 mg/dL	15 (32.6)	1 (25)	
Urine albumin	Present	36 (78.2)	1 (25)	0.020
	Absent	10 (21.8)	3 (75)	
X-ray changes	Present	38 (82.6)	4 (100)	0.76
	Absent	8 (17.4)	0	

**Figure 1:**
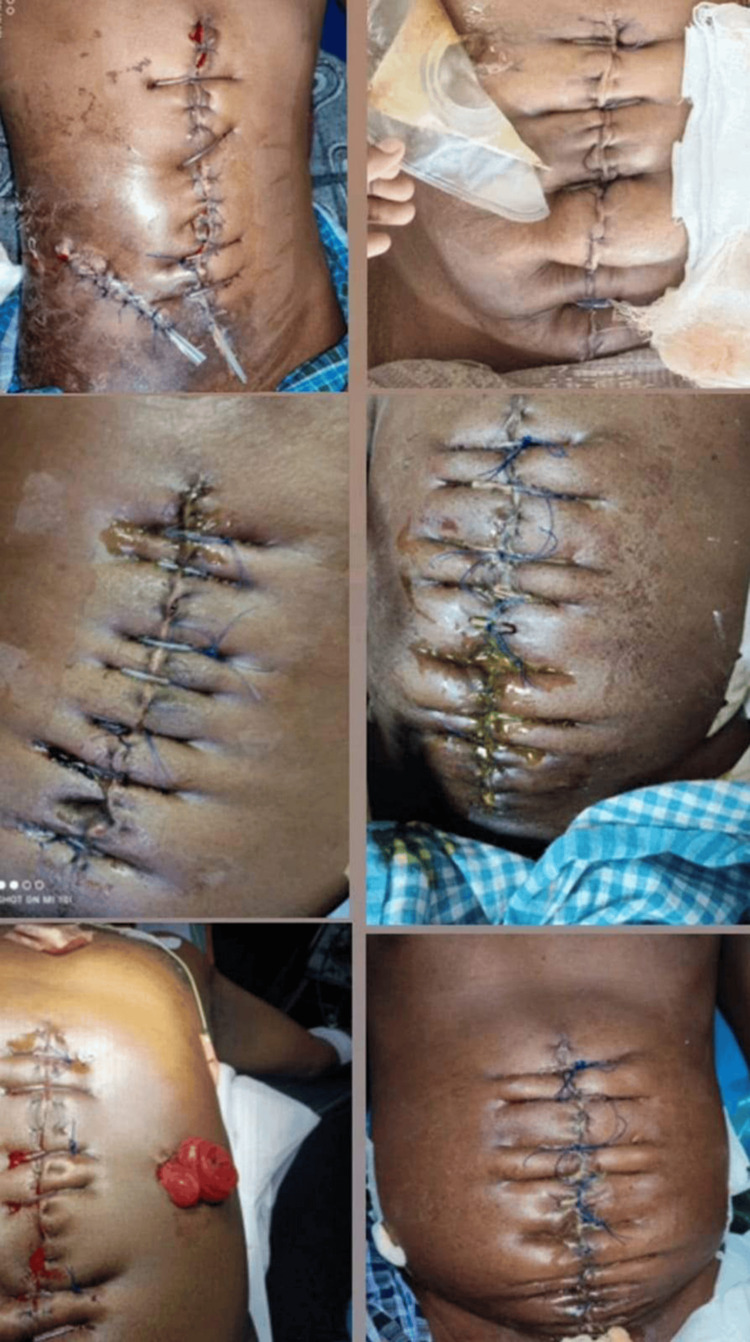
Postoperative pictures of surgical site infection Surgical site infection was associated with the type of surgery and operating time, with significant p values of 0.004 and 0.03, respectively.

About 78.3% and 75% of the patients who underwent emergency and elective procedures developed wound dehiscence on PODs 5-10, respectively, while others developed dehiscence within five PODs.The POD of wound dehiscence was significantly associated with the type of surgery, with a p-value of 0.032 by Fischer’s exact test, as evident from Table 4. SSI is an important post-op complication following any surgery. If SSI is not treated adequately, this may lead to wound dehiscence (Figure 1). SSI (postoperative complication) was associated with the type of surgery and operating time, with significant p values of 0.004 and 0.03, respectively, whereas the type of incision was not associated with SSI (Table 5).

**Table 4:** Association between the day of wound dehiscence and the type of surgery (n=50) n=number of study participants Fischer’s exact test was done, and a p-value <0.05 was considered significant.

Wound dehiscence	Type of surgery		P value
	Emergency, n (%)	Elective, n (%)	
POD 0-5	10 (21.7)	1 (25)	0.032
POD 5-10	36 (78.3)	3 (75)	

**Table 5:** Association between SSI and surgical factors (n=50) n=number of study participants. Fischer’s exact test was done, and p value <0.05 was considered significant.

Surgical factors	SSI		P value
	Present, n (%)	Absent, n (%)	
Type of surgery			
Emergency	7 (70)	39 (97.5)	0.004
Elective	3 (30)	1 (2.5)	
Surgical wounds classification			
Clean	4 (40)	15 (37.5)	0.343
Clean contaminated	6 (60)	18 (45)	
Contaminated	0	7 (17.5)	
Operating time			
<2 hour	2 (20)	11 (27.5)	0.03
2-4 hour	4 (40)	26 (65)	
>4 hour	4 (40)	3 (7.5)	

## Discussion

Acute wound failure is one of the major complications following laparotomy with significant morbidity and mortality. Several factors affect outcomes in different centers, including patient factors and hospital factors. Patient factors are demographics, factors related to presentation, and etiology. The quality of a hospital depends on various factors, such as its infrastructure, attending personnel, and the volume of workload it handles. The higher incidence of wound dehiscence in our study may be due to an increased number of emergency surgeries.

Our study shows that mass closure of post-laparotomy wound dehiscence is more common among the age group 41-50 years, followed by 61-70 years. Gejoe et al. found that emergency laparotomies were common in the age group 40-80 years [10]. Delayed wound healing in the elderly is associated with an altered inflammatory response, such as delayed T-cell infiltration into the wound area with alterations in chemokine production and reduced macrophage phagocytic capacity [11]. Chalya et al. found a higher prevalence of males than females (male-to-female ratio of 2.8:1) among 872 patients who underwent midline laparotomy similar to our study [12]. In our study, 88% of patients with wound dehiscence underwent emergency surgery, which is similar to the findings of Riou et al. [6]. They found that 16 (51.6%) wound dehiscence out of 31 were following emergency surgeries. Gejoe et al. found the common etiologies to be perforation in 57% of the cases and obstruction in 37% of the cases. In our study, we found that obstruction occurred more frequently than perforation, with 36% of cases presenting with obstruction and 30% with perforation [10].

Comorbidities are associated complications of wound healing after surgical procedures. Obese patients are more prone to SSI. Complications may occur due to reduced blood flow and oxygen supply in the fatty tissue beneath the skin. In addition, adipose tissue may complicate the delivery of antibiotics. Obese patients often experience increased tension on wound edges, which can lead to wound dehiscence. Wound tension increases tissue pressure, reducing microperfusion and the availability of oxygen [13]. Hyperglycemia can also add to the oxidative stress when the production of reactive oxygen species (ROS) exceeds the antioxidant capacity. The formation of advanced glycation end products (AGEs) under hyperglycemia and the interaction with their receptors (RAGE) are associated with impaired wound healing. However, diabetes mellitus was not associated with wound dehiscence in our study population [14]. Among the 15 diabetic patients, 10 were known cases preoperatively, and five cases were detected incidentally whose random blood sugar (RBS) was above 200 mg/dL and urine albumin was positive. Patients who underwent midline redo-laparotomy for obstruction had a higher incidence of wound dehiscence, resulting in delayed wound healing after suture removal on POD 20.

Our study found an association between abdominal wound dehiscence following laparotomy and bleeding and clotting time among participants. About 35% of the patients presented abnormal clotting times, and 75% of these cases were caused by malignancy. Patients with cardiovascular risk factors, including arterial hypertension, also exhibit unfavorable fibrin clot characteristics. Based on clinical phenotypes of some dysfibrinogenaemias, abnormal fibrin clot features have been suggested to play a role in defective wound healing. However, the exact relationships between fibrin clot properties assessed in plasma, bleeding time, and clotting time on wound healing in human subjects remain unclear. Loose fibrin fiber networks may prolong bleeding time and slow skin wound healing [15].

Patients who smoked and had pre-existing comorbidity such as COPD showed a delay in wound healing. There was an increase in a variety of complications such as infection, wound rupture, anastomotic leakage, wound and flap necrosis, and epidermolysis and a decrease in the tensile strength of wounds of those who had a history of smoking [16-18]. Inoue et al. found that smoking cessation for four weeks before surgery can reduce the duration of hospital stay and the rate of suture failure [19]. Kenig et al. reported no significant differences between the study and control groups regarding anemia, which is synonymous with our study. Similar findings were reported by Talukdar et al. [20]. However, van Ramshorst et al. reported that 33% of cases were uremic [3].

In our study, out of 50 cases operated on, albuminuria was present in 74% (n=37) of the patients during their postoperative hospital stay due to sepsis causing hypoalbuminemia. It is a risk factor for a burst abdomen. Choudhury et al. reported that 76.79% of their patients with wound dehiscence had hypoalbuminemia [21]. Jaiswal et al. reported that 58% of cases with burst abdomen had hypoproteinemia with serum total proteins less than 6 g% [22].

Results of our study showed that there is an association between SSI and the type of surgery among study participants. In our study, a total of 10 cases had developed surgical site infection; seven patients developed superficial SSI, and two patients developed SSI with dehiscence of the abdominal rectus. The study by Carlson et al. on 1,072 patients who underwent abdominal surgery with midline incision found that the type of incision was not associated with wound complications after a mean follow-up of 22 months, which is similar to our study [23].

Spiliotis et al. followed 3,500 patients who underwent surgical procedures and found that 15 patients developed wound dehiscence. The majority of the procedures of our study were emergency procedures (92%, n=46), while the remaining constituted elective procedures (8%, n=4). This could be due to recent advancements in surgical techniques and strict aseptic precautions, including postoperative care [24].

Riou et al. found that wound dehiscence following laparotomy was associated with considerable mortality [6], whereas the current study had a mortality of 4% (two out of 50 participants deceased) following treatment for abdominal wound dehiscence. Modi et al. reported a mortality rate as high as 45% following abdominal wound dehiscence [25].

Since the study was conducted in a selected tertiary care hospital that attracted people from a particular geographical area, there are certain limitations to consider when dealing with selection bias. Moreover, the study was conducted in a tertiary care hospital that may receive referral from secondary-level hospitals and private hospitals, so there is a chance of complicated surgeries being diverted to the study setting, which might exaggerate the occurrence of wound dehiscence. We also failed to acquire data on previous history of abdominal surgeries and its association with wound dehiscence as repeated surgeries can cause adhesions and complicate the surgical anatomy leading to complications in the perioperative and postoperative period. The expertise of the operating surgeon also plays a role that could not have been assessed.

## Conclusions

Abdominal wound dehiscence is an arduous and challenging postoperative complication that necessitates immediate intervention. The type of surgery and operating time were associated with SSI. Strict postoperative care emphasizes reducing the risk of wound infection and other factors related to wound dehiscence.
